# Supplementary material for: Design, Synthesis, and Biological Evaluation of N-Acylhydrazones and Their Activity Against Leishmania amazonensis Promastigotes
Source: Microorganisms. 2025 Jul 2;13(7):1563. doi: 10.3390/microorganisms13071563 (PMC12298860; doi:10.3390/microorganisms13071563)
Supplement: Supplementary file 1 [file microorganisms-13-01563-s001.zip › HPLC amostras-20241114T144635Z-001.zip!/HPLC amostras/HPLC composto 34 - PMM 12-18.pdf]

Farmanguinhos - Fiocruz
Serviço de Métodos Analíticos

PMM 12-18 R Rep.1.lcd
2018 - Síntese 1 - João Mafra - 2-8/2-11-5 - PMM 12-18 R Rep.1.lcd
31/07/2018, 15:25:04

Sample Name : PMM 12-18 R Rep.1
Sample ID : 121165
Sample Full Path : 2018 - Síntese 1 - João Mafra - 2-8/2-11-5 - PMM 12-18 R Rep.1.lcd
Data Filename : PMM 12-18 R Rep.1.lcd
Method Filename : Nitroimidazol.lcm
Batch File : 2018 - Síntese 1 - João Mafra - CLAE-11 - CLAE 11_2018 _0731.lcb
Vial # : 1-5 Sample Type : Unknown
Injection Volume : 10 uL
Date Acquired : 31/07/2018 15:25:32 Acquired by : Licinio Fontoura
Date Processed : 01/08/2018 10:35:41 Processed by : Licinio Fontoura
Data Comment :

Preparação da amostra

A 1,04mg de amostra foram adicionados 3000µL de metanol, agitado em vortex e levada ao ultrasom por 1 minuto (Conc.de 0,35mg/mL).
Posteriormente foi filtrada em membrana PVDF de 0,22µm e injetada.

Method

<<Header>>

<<Pump>>
Total Flow : 1,0000 mL/min
Pump B : LC-20ADXR
Pump A : LC-20ADXR

<<Mobile Phase Name>>
Pump A Mobile Phase A : Água
Pump B Mobile Phase A : Acetonitrila

<<LC Time Program>>

Time	Module	Command	Value	Comment
0,01	Pumps	Pump B Conc.	20	
5,00	Pumps	Pump B Conc.	20	
22,00	Pumps	Pump B Conc.	90	
25,00	Pumps	Pump B Conc.	90	
33,00	Pumps	Pump B Conc.	20	
39,00	Pumps	Pump B Conc.	20	
39,01	Controller	Stop		

<<Data Acquisition>>
LC Stop Time : 39,01 min

<<Autosampler>>
Cooler Temperature : Off

<<Oven>>
Oven Temperature : 30 C

<<Column Infomation>>
Column Name : Supelcosil LC-8 5µm
Column ID : CL0173
Column Length : 150 mm
Inner Diameter : 4,6 mm

Instrumentation

<<Instrument>>

<<Pump A>>
Pump A Type : LC-20ADXR

<<Pump B>>
Pump B Type : LC-20ADXR

<<System Controller>>
SCL Type : CBM-20A

<<Autosampler>>
Autosampler : SIL-30AC

<<Oven>>
Oven Type : CTO-20AC

<<Column>>
Column Name : Supelcosil LC-8 5µm
Column ID : CL0173
Length : 150 mm
Internal Diameter : 4,6 mm
Max Number of Injections : 100000
Expiration Date : 29/04/2025
Max Temperature : 60,0 C
Installation Date : 14/03/2018

<<PDA>>
Detector Name : PDA
Detector Type : SPD-M20A

<<Degasser>>
Degasser : DGU-20A5R

Farmanguinhos - Fiocruz
Serviço de Métodos Analíticos

PMM 12-18 R Rep.1.lcd
2018 - Síntese 1 - João Mafra - 2-8/2-11-5 - PMM 12-18 R Rep.1.lcd
31/07/2018, 15:25:04

Chromatogram

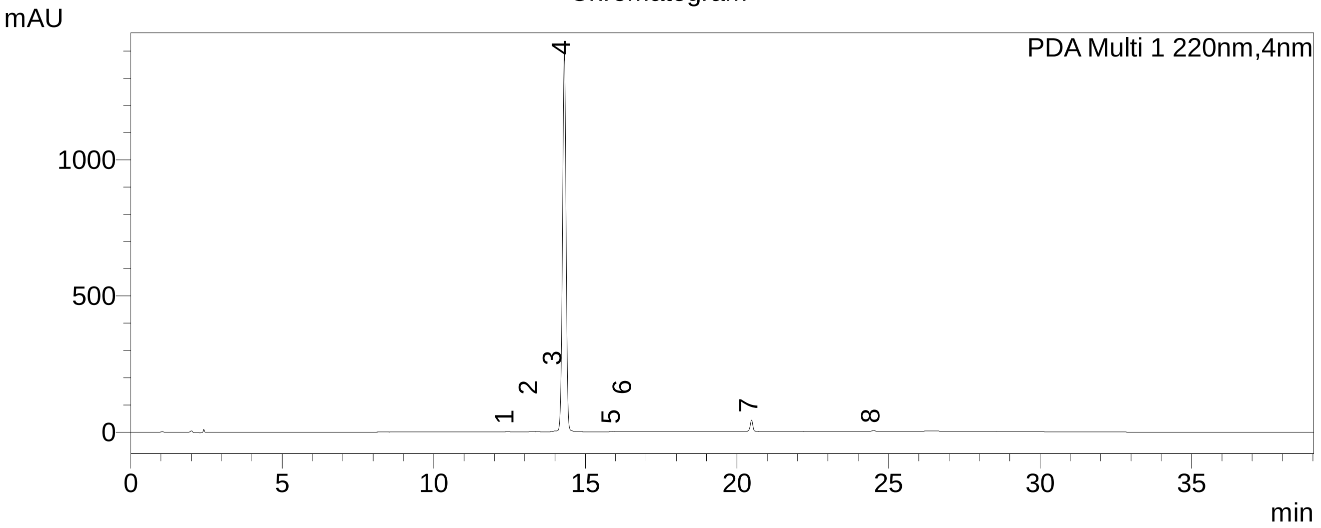

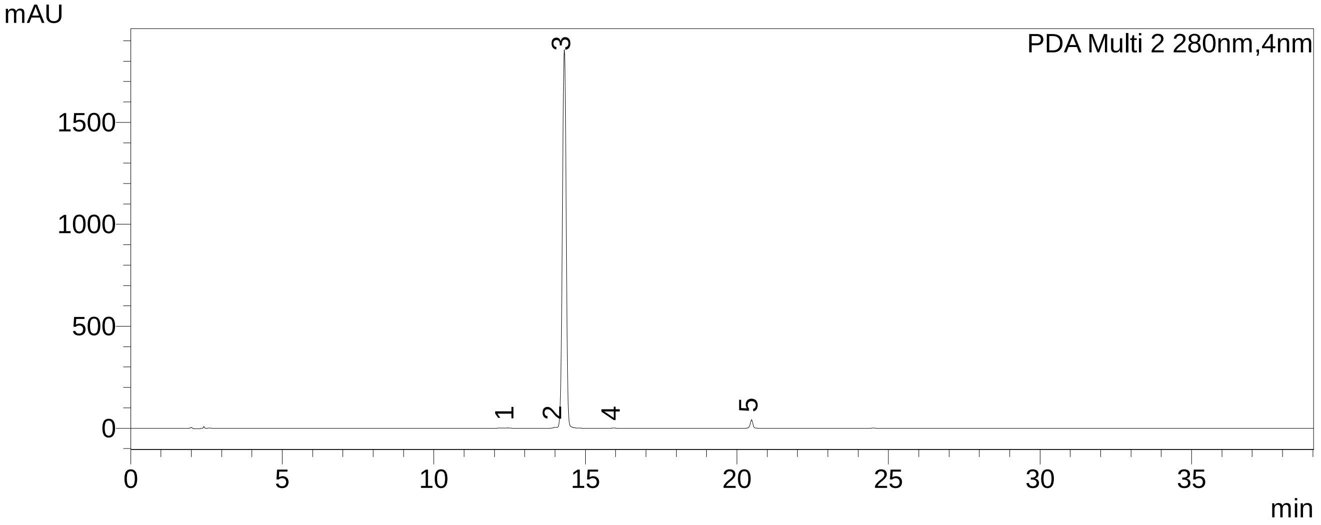

Peak Table

PDA Ch1 220nm

Peak#	Ret. Time	Name	Area	Area%	Theoretical Plates/meter(USP)	Tailing Factor	Resolution(USP)	Capacity Factor(k')
1	12,44		14395	0,1	287147	1,039	--	--
2	13,22		9731	0,1	144401	--	2,589	0,062
3	14,02		25752	0,2	66117	--	1,731	0,126
4	14,30		11435643	97,0	405070	0,965	0,725	0,150
5	15,94		12540	0,1	535864	--	7,172	0,281
6	16,32		9636	0,1	54857	--	0,797	0,312
7	20,49		263905	2,2	1367028	0,922	9,259	0,647
8	24,51		15094	0,1	2272347	0,965	23,043	0,970
Total			11786696	100,0				

PDA Ch2 280nm

Peak#	Ret. Time	Name	Area	Area%	Theoretical Plates/meter(USP)	Tailing Factor	Resolution(USP)	Capacity Factor(k')
1	12,44		13469	0,1	305490	1,077	--	--
2	14,01		34883	0,2	121251	--	4,851	0,126
3	14,30		16322356	98,1	372650	0,961	0,885	0,150
4	15,94		9988	0,1	646705	1,006	7,327	0,281
5	20,49		259869	1,6	1368580	0,924	23,586	0,647

Farmanguinhos - Fiocruz
Serviço de Métodos Analíticos

PMM 12-18 R Rep.1.lcd
2018 - Síntese 1 - João Mafra - 2-8/2-11-5 - PMM 12-18 R Rep.1.lcd
31/07/2018, 15:25:04

Peak#	Ret. Time	Name	Area	Area%	Theoretical Plates/meter(USP)	Tailing Factor	Resolution(USP)	Capacity Factor(k')
Total			16640565	100,0				
